# Impact of maternal depression and anxiety on immunization status of children: a prospective cohort study

**DOI:** 10.1186/s13690-024-01323-3

**Published:** 2024-06-17

**Authors:** Shannon E. MacDonald, Manisha Dhungana, Victoria Stagg, Sheila McDonald, Deborah McNeil, James D. Kellner, Suzanne Tough, Vineet Saini

**Affiliations:** 1 Faculty of Nursing, University of Alberta, Edmonton, Canada; 2 Department of Pediatrics, Cumming School of Medicine, University of Calgary, Calgary, Canada; 3 Research and Innovation, Public Health Evidence and Innovation, Alberta Health Services, Calgary, Canada; 4 Department of Community Health Sciences, Cumming School of Medicine, University of Calgary, Calgary, Canada

**Keywords:** Maternal, Depression, Anxiety, Immunization, Vaccine

## Abstract

**Background:**

Maternal depression and anxiety can have a detrimental impact on birth outcomes and healthy child development; there is limited knowledge on its influence on immunization schedule adherence. Therefore, the objectives of this study were to determine the impact of maternal depression and anxiety in the perinatal period on prolonged vaccine delay of childhood vaccines.

**Methods:**

In this prospective cohort study, we analyzed linked survey and administrative data of 2,762 pregnant women in Calgary, Alberta, Canada. Data were collected at two time-points: prenatal (< 25 weeks of gestation) and postpartum (4 months postpartum). We used multivariable logistic regression to examine the association between depression and anxiety with prolonged immunization delay, adjusting for covariates.

**Results:**

In multivariable analysis, maternal depression at either time point was not associated with prolonged delay for DTaP-IPV-Hib (OR 1.16, 95% CI 0.74–1.82), MMR/MMRV (OR 1.03, 95% CI 0.72–1.48), or all routine childhood vaccines combined (OR 1.32, 95% CI 0.86–2.04). Maternal anxiety at either time point was also not associated with prolonged delayed for DTaP-IPV-Hib (OR 1.08, 95% CI 0.77–1.53), MMR/MMRV (OR 1.07, 95% CI 0.82–1.40), or all vaccines combined (OR 1.00, 95% CI 0.80–1.26). In both the depression and anxiety models, children of Canadian-born mothers had higher odds of prolonged delay, as did those with low-income mothers.

**Conclusion:**

Health care providers can be reassured that maternal depression and anxiety do not appear to influence maternal commitment to routine immunization. Findings suggested that low income and household moves may influence adherence to vaccine schedules and health care providers may want to provide anticipatory guidance to these families.

**Supplementary Information:**

The online version contains supplementary material available at 10.1186/s13690-024-01323-3.

**Table:** 

**Text box 1. Contributions to the literature**
• Maternal perinatal depression and anxiety can have a detrimental impact on birth outcomes and healthy child development, but there is limited knowledge on its influence on uptake of childhood vaccines
• Perinatal depression and/or anxiety was not found to be associated with prolonged delay for most early childhood vaccines
• Reassuring/negative findings can be useful to guide clinicians and policy-makers in their decision of where to focus scarce resources for targeted immunization efforts.

## Introduction

Depression and anxiety during the prenatal and postpartum period can have a detrimental impact on birth outcomes and healthy child development [1, 2]. In particular, maternal mental health challenges in the perinatal period may result in reduced healthcare-seeking behaviours for children, including uptake of preventive health care [3–5].

Childhood immunization is an essential and cost-effective preventive health intervention to protect children from vaccine-preventable diseases [6, 7]. Complete and timely immunization with routinely scheduled childhood vaccines is important to ensure optimal protection from vaccine-preventable diseases [8]. Thus, missed or delayed immunization increases risk for severe infections, such as pertussis and measles, in young children [9].

There is limited knowledge on the impact of maternal depression or anxiety on immunization status of children [3, 4, 10–13] and very few studies have assessed both prenatal and postpartum depression and anxiety [3, 14]. The existing literature shows mixed evidence of the relationship between maternal depression or anxiety and complete immunization of the child, and most results come from studies with very small sample sizes [4, 11–13]. In addition, no previously published studies have assessed the *timeliness* of immunization, defined as lack of delays in administration of vaccine(s) according to the recommended vaccine schedule, in this population. This study addresses these gaps in knowledge. Specifically, we aimed to examine the association between prenatal and postpartum *depression*, and timeliness of immunization for (a) diphtheria, tetanus, acellular pertussis, polio and *Hemophilus influenzae* type b (DTaP-IPV-Hib) vaccine, (b) measles, mumps and rubella (MMR)/MMR-Varicella (MMRV) vaccines, and (c) for all vaccines in the recommended immunization schedule combined. We also aimed to examine the association between prenatal and postpartum *anxiety*, and timeliness of immunization for DTaP-IPV-Hib, MMR/MMRV, and all vaccines in the recommended immunization schedule combined.

## Methods

### Study design, population, and setting

The data for this study was obtained from an existing prospective pregnancy cohort study, the “All Our Families” (AOF) study. AOF recruited 3387 pregnant women in Calgary, Alberta, Canada, a city of 1.27 million people, between May 2008 and December 2010 [15]. The detailed recruitment process for AOF has been described previously [16]. Two surveys were completed by the cohort in the prenatal period (before 25 weeks and at 34–36 weeks), with six surveys after birth (at 4 months, 1,2,3,5, and 8 years). Women who consented to linkage of their survey data with administrative health records through their personal health number (PHN) provided at the time of recruitment (*N* = 2855, 84%) were included in this analysis.

For the purpose of this analysis, we drew data from surveys at two time points: *prenatal*, defined as < 25 weeks of gestation (*N* = 2,849) and *postpartum*, defined as 4 months postpartum (*N* = 2,699). The number of participants who were sent surveys varied slightly by time point. Self-reported demographic and other characteristics from the surveys (including data from self-administered depression and anxiety scales) were linked using unique identifiers to public health immunization data [17].

### Exposures

#### Depression

Depression symptoms were measured using the Edinburgh Postpartum Depression Scale (EPDS), a 10- item self-report validated instrument used to screen women for depression in their postpartum period [18]. This tool is also used to detect early prenatal depression [19]. The maximum score for both prenatal and postpartum periods is 30 and we used the standard cut off score of ≥ 13 that indicates greater risk of depression [16]. The sensitivity of the EPDS for identifying women with major or minor depression, as diagnosed according to the Research Diagnostic Criteria (RDC), was found to be 86%, while the specificity was 78% [18]. We created a categorical variable for depression with two levels: (a) prenatal and/or postpartum depression and (b) no depression at either time point.

#### Anxiety

Anxiety was measured using the Spielberger State Anxiety Scale, a self-report validated questionnaire of twenty questions that was used to measure the mothers’ state of anxiety [20]. The scale ranges from 20 to 80, and the standard cut off score of ≥ 40 indicates an anxious state in pregnant women, with 81% sensitivity and 79.8% specificity [21]. We created a categorical variable for anxiety with two levels: (a) prenatal and/or postpartum anxiety and (b) no anxiety at either time point.

### Outcomes

#### Vaccine delay

The AOF survey data were linked to public health administrative databases (Phantim and Medipatient) using a combination of maternal PHN, and maternal and child date of birth [22]. These public health databases contain records of all vaccines administered to children as part of Alberta’s publicly funded immunization program. Through this program, all childhood vaccines are administered by public health nurses and entered into these immunization registries. Data are routinely audited to ensure accuracy of data entry. For the purpose of this study, we linked AOF survey data to vaccine events (vaccine type, dose, and date of administration) for children up to 24 months of age (*N* = 2763 children) (Supplementary Fig. 1) [22]. Later, one child was dropped from the linked database due to invalid age information thereby leaving 2762 observations for the analysis.

Using the method developed by Luman et al. [23], we calculated delay in administration of all vaccine doses (combined) in the recommended routine childhood immunization schedule by 2 years of age (see Table 1), as well as for two individual vaccines: a multi-dose vaccine, DTaP-IPV-Hib, and a single dose vaccine, MMR/MMRV. ‘Delayed immunization’ was defined as doses administered after the end of a grace period, which was the maximum number of days before the child increased in age to be the next month old. It was measured as ‘Days delayed’ and calculated as the cumulative number of days delayed after the grace period for all recommended vaccines of one or more doses, assessed at 2 years of age. For instance, the recommended age for administering the first dose of DTaP-IPV-Hib is at 2 months and the grace period ends at 3 months of age (90–92 days, depending on month). If a child received their first dose of DTaP-IPV-Hib 1 day after the grace period i.e., at 93 days, then it is counted as 1 day of delay for the first dose of DTaP-IPV-Hib vaccine. For multi-dose vaccines, days delayed was calculated by adding up days delayed for each dose. For example, suppose a child received the first dose of DTaP-IPV-Hib vaccine at 110 days (3 ½ months), the 2nd dose at 180 days (6 months) and the 3rd dose at 330 days (11 months). Then, the days delayed would be considered to be from days 93 to 109 for first dose, from days 154 to 179 for 2nd dose, and from days 216 to 329 for the 3rd dose. Thus, the days delayed for the DTaP-IPV-Hib vaccine would be 157 days, i.e., the cumulative delay for the 3 doses = (110 − 93) + (180 − 154) + (330 − 216).

**Table 1:** Alberta routine childhood immunization schedule at the time of the study (2008–2012)

Vaccines	2 months	4 months	6 months	12 months	18 months	Total Doses
**January 1 2008 - June 30 2010**						
Diphtheria, Tetanus, Acellular Pertussis, Polio, *Haemophilus influenzae type b* (DTaP-IPV-Hib)	X	X	X		X	4
Pneumococcal Conjugate (PCV 7)	X	X	X		X	4
Meningococcal Conjugate (Men-C)	X	X		X		3
Measles, Mumps, Rubella (MMR)				X		1
Varicella (V)				X		1
**July 1 2010 – December 31 2012** ^a^						
Diphtheria, Tetanus, Acellular Pertussis, Polio, *Haemophilus influenzae type b* (DTaP-IPV-Hib)	X	X	X		X	4
Pneumococcal Conjugate (PCV 13)	X	X		X		3
Meningococcal Conjugate (Men-C)	X	X		X		3
Measles, Mumps, Rubella, Varicella (MMRV)				X		1

^a^ On July 1 2010, the pneumococcal conjugate vaccine changed from PCV 7 to PCV 13. On September 1 2020 the measles, mumps, rubella vaccine was combined with the varicella vaccine to the create the MMRV vaccine, but no change in timing of number of doses occurred. The other vaccines and their administration schedules remained the same

We then calculated our primary outcome, ‘prolonged delay’, which we defined as delayed for more than 6 months (≥ 7 months) for all vaccines combined in the schedule, or the multi-dose vaccine, DTaP-IPV-Hib, or the single dose vaccine, MMR/MMRV. Thereafter, we assessed the association of the primary exposures of maternal depression and anxiety with prolonged delay in immunization for all routine childhood immunizations combined and separately for DTaP-IPV-Hib and MMR/MMRV immunizations.

### Covariates

Various covariates such as parity, maternal age in years at the time of delivery, maternal marital status, born in Canada, maternal education, income, social support, number of household moves, and gestational term of baby were examined for potential inclusion in the models based on association with delayed or incomplete vaccination in the existing literature [24–31]. All covariates were obtained during the survey at the 1st trimester of the pregnancy, except maternal age, which was obtained at the time of delivery.

### Statistical analysis

To describe the characteristics of the sample, we calculated means and standard deviations (SD) for the normally distributed continuous variables, and medians and interquartile range (IQR) for continuous variables that were not normally distributed. For categorical variables, we calculated frequencies and percentages.

To examine the association between the exposures/covariates and prolonged delay of immunization, we conducted bivariate and multivariable analyses. For the binary outcome (0 = no prolonged delay; 1 = prolonged delay), we used logistic regression. No prolonged delay was defined as delay between 0 and 215 days and prolonged delay was defined as delay ≥ 216 days; 215 days is the maximum number of days that reflects 6 months + 30 days (just prior to turning 7 months old). We ran two logistic regression models, one with depression as the primary exposure and second with anxiety as the primary exposure.

Covariates found to be significantly related to the relevant prolonged delay outcome (DTaP, MMR, or All Vaccines) in bivariate regression (defined as p < = 0.25) were included in the full models. Backward elimination was used to determine the most parsimonious model, with the primary exposure variables (prenatal and/or postpartum depression, prenatal and/or postpartum anxiety) retained in the relevant models throughout the process of backward elimination. Two-way interaction terms were tested for each combination of explanatory variables remaining in the full model, and tested for significance using the likelihood ratio test. Following the process of backward elimination and the selection of a “final” model of covariates, all explanatory (independent) variables that were dropped from the model during the steps of backward elimination were checked for their qualification as a confounder. Any that qualified (changed the coefficient of the exposure variable by ≥ 10%) were retained in the final model.

### Sensitivity analysis

As part of a sensitivity analysis, we assessed depression and anxiety separately at two different time points, i.e. prenatal (< 25 weeks gestation) and postpartum (4 months post-partum). The intent was to understand if the timing of depression and anxiety symptoms assessment was associated with immunization delay.

Ethical approval for this study was received from the Conjoint Health Research Ethics Board at the University of Calgary (REB 14–0925).

## Results

Of 2,762 participants, 266 (9.63%) and 581 (21.04%) mothers had depression and anxiety symptoms, respectively (Table 2). Most mothers were married or in a common-law relationship (94.61%), born in Canada (77.73%), had some or completed post-secondary education (89.10%), had income greater than $60,000 (80.30%), a history of no household moves (75.74%), and had high social support (72.77%).

**Table 2:** Characteristics of participants (*N* = 2762)

Variable	Number (%)
**Depression**	
Prenatal depression Postpartum depression Prenatal &/or postpartum depression	196 (7.10) 134 (4.85) 266 (9.63)
**Anxiety**	
Prenatal anxiety Postpartum anxiety Prenatal &/or postpartum anxiety	420 (15.21) 374 (13.54) 581 (21.04)
**Parity**	
No previous birth Previous birth	1365 (49.42) 1366 (49.46)
**Marital status**	
Married/common law Others	2613 (94.61) 135 (4.89)
**Born in Canada**	
Yes No	2147 (77.73) 604 (21.87)
**Education**	
High school or less Some or completed post-secondary	286 (10.35) 2461 (89.10)
**Income**	
Less than $60,000 $60,000 or more	445 (16.11) 2218 (80.30)
**Social support**	
Low High	570 (20.64) 2010 (72.77)
**Number of household moves**	
None ≥ 1	2092 (75.74) 521 (18.86)
**Gestational age**	
≥ 37 weeks ≤ 36 weeks	2385 (86.35) 208 (7.53)

In bivariate analysis (Table 3), neither maternal depression nor maternal anxiety (at either the prenatal and /or postpartum periods) increased the likelihood of a child having a prolonged delay for DTaP-IPV-Hib, MMR/MMRV, or all routine childhood vaccines combined. Some specific covariates, such as parity, country of birth, income, and number of household moves, were associated with the outcome of prolonged delay.

**Table 3:** Odds ratio (OR) for bivariate associations between primary exposures (depression, anxiety), covariates, and outcome (prolonged delay) in DTaP-IPV-Hib, MMR/MMRV, and all vaccines combined, among two year old children in Calgary, Alberta (*N* = 2762)

Variables	*DTaP-IPV-Hib* OR (95% CI)	*p*-value	*MMR/MMRV* OR (95% CI)	*p*-value	*All vaccines combined* OR (95% CI)	*p*-value
**Prenatal and/or postpartum depression** (ref: No depression)	1.42 (0.94–2.13)	0.093	1.02 (0.72–1.46)	0.895	1.05 (0.78–1.41)	0.767
**Prenatal and/or postpartum anxiety** (ref: No anxiety)	1.18 (0.86–1.63)	0.302	1.06 (0.82–1.38)	0.638	1.04 (0.83–1.29)	0.756
**Multi-parity** (ref: Primiparous)	1.54 (1.18–2.02)	0.002	1.20 (0.97–1.48)	0.097	1.35 (1.13–1.62)	0.001
**Maternal age at delivery** (in years)	0.98 (0.95–1.01)	0.241	0.98 (0.96–1.01)	0.218	0.98 (0.96–1.00)	0.115
**Single marital status** (ref: Married/ common law)	0.82 (0.43–1.59)	0.567	1.13 (0.71–1.81)	0.601	0.91 (0.60–1.39)	0.675
**Born in Canada** (ref: No)	1.22 (0.87–1.70)	0.247	1.51 (1.14–1.99)	0.004	1.29 (1.03–1.61)	0.027
**Post-secondary education** (ref: No Post-secondary)	0.63 (0.43–0.93)	0.018	0.87 (0.63–1.21)	0.416	0.79 (0.60–1.05)	0.104
**Income ≥ $60,000** (ref: <60,000)	0.58 (0.42–0.79)	0.001	0.73 (0.56–0.95)	0.021	0.72 (0.57–0.90)	0.005
**High social support** (ref: Low)	1.11 (0.79–1.56)	0.565	1.01 (0.78–1.31)	0.947	0.97 (0.78–1.20)	0.765
**≥ 1 household moves** (ref: None)	1.48 (1.08–2.03)	0.015	1.21 (0.93–1.57)	0.152	1.28 (1.03–1.60)	0.028
**Preterm** (ref: >=37 wks)	1.31 (0.82–2.09)	0.250	0.93 (0.62–1.39)	0.714	0.93 (0.66–1.30)	0.659

### Multivariable association between depression and prolonged delay of DTaP-IPV-Hib, MMR/MMRV, and all vaccines combined

After controlling for covariates, maternal depression at either time point (prenatal and /or postpartum) did not increase the likelihood of a child having a prolonged delay for DTaP-IPV-Hib (OR 1.16, 95% CI 0.74–1.82), MMR/MMRV (OR 1.03, 95% CI 0.72–1.48), or all routine childhood vaccines combined (OR 1.32, 95% CI 0.86–2.04) (Table 4).

In the multivariable depression model, some covariates were associated with prolonged delay. Children of multiparous mothers had higher odds of prolonged delay for DTaP-IPV-Hib (OR 1.64, 95% CI 1.22–2.20), and all vaccines combined (OR 1.48, 95% CI 1.21–1.81). Children of mothers born in Canada had higher odds of prolonged delay for MMR/MMRV (OR 1.58, 95% CI 1.17–2.13) and all routine childhood vaccines combined (OR 1.36, 95% CI 1.06–1.73).

Notably for DTaP-IPV-Hib immunization, there were interaction effects for some covariates on prolonged delay (Table 4, Supplementary Figs. 2–5), especially among mothers who were born in Canada and moving households. Preterm babies of such mothers had higher odds of prolonged delay of DTaP-IPV-Hib immunization compared to mothers of full term babies (OR = 3.87, 95% CI: 1.69–8.85, Table 4, Supplementary Fig. 5).

**Table 4:** Final multivariable logistic regression models depicting adjusted odds ratio (aOR) for association between maternal depression and prolonged delay in DTaP-IPV-Hib, MMR/MMRV and all vaccines combined among children in Calgary, Alberta

	DTaP-IPV-Hib *N* = 2463		MMR/MMRV *N* = 2503		All vaccines combined *N* = 2481	
Variable	aOR (95% CI)	*p*-value	aOR (95% CI)	*p*-value	aOR (95% CI)	*p*-value
Prenatal &/or postpartum depression*	1.16 (0.74–1.82)	0.530	1.03 (0.72–1.48)	0.877	1.32 (0.86–2.04)	0.209
Preterm	0.14 (0.02–1.12)	0.064	-**		-	
Parity	1.64 (1.22–2.20)	0.001	-		1.48 (1.21–1.81)	0.000
Born Canada	1.25 (0.84–1.84)	0.272	1.58 (1.17–2.13)	0.003	1.36 (1.06–1.73)	0.014
High income	0.46 (0.30–0.71)	0.000	0.67 (0.50–0.90)	0.008	0.74 (0.57–0.96)	0.022
Moves	0.54 (0.26–1.13)	0.102	-		1.29 (1.02–1.63)	0.032
***Interaction terms***						
Born Canada X Preterm***	8.75 (1.08–70.96)	0.042	-		-	
Not Preterm (37 + Wks): Born in Canada versus Outside	1.25 (0.84–1.84)	0.272	-		-	
Preterm ( < = 36 Wks): Born in Canada versus Outside	10.90 (1.39–85.4)	0.023	-		-	
High income X Moves***	2.90 (1.29–6.52)	0.010	-		-	
Moves None: High Income versus Low Income	0.46 (0.30–0.71)	0.000	-		-	
Moves > = 1: High Income versus Low Income	1.34 (0.67–2.70)	0.404	-		-	
Moves X Preterm***	3.20 (1.13–9.01)	0.028	-		-	
Born Outside Canada Moves None: Preterm versus Not Preterm	0.14 (0.02–1.12)	0.064	-		-	
Born Outside Canada Moves > = 1: Preterm versus Not Preterm	0.44 (0.05–3.57)	0.444	-		-	
Born Within Canada Moves None: Preterm versus Not Preterm	1.21 (0.63–2.34)	0.569	-		-	
Born Within Canada Moves > = 1: Preterm versus Not Preterm	3.87 (1.69–8.85)	0.001	-		-	
*Depression X Parity*					0.53 (0.28–0.99)	0.045
Primips: Depression versus No Depression					1.32 (0.85–2.04)	0.209
Multips: Depression versus No Depression					0.69 (0.44–1.10)	0.118

* Prenatal (< 25 weeks gestation) and/or postpartum (4 months post-partum) depression

** Variables were not statistically significant in the model and therefore were excluded during the backward elimination process

*** Interaction between explanatory variables

### Multivariable association between anxiety and prolonged delay of DTaP-IPV-Hib, MMR, and all vaccines combined

Similar to depression, prenatal and/or postpartum anxiety did not increase the likelihood of a child having a prolonged delay for DTaP-IPV-Hib (OR 1.08, 95% CI 0.77–1.53), MMR/MMRV (OR 1.07, 95% CI 0.82–1.40), and all vaccines combined (OR 1.00, 95% CI 0.80–1.26) after controlling for covariates (Table 5).

**Table 5:** Final multivariable logistic regression models depicting adjusted odds ratio (aOR) for association between maternal anxiety and prolonged delay in DTaP-IPV-Hib, MMR and all vaccines combined among children in Calgary, Alberta

	DTaP-IPV-Hib *N* = 2313		MMR *N* = 2330		All vaccines combined *N* = 2330	
Variable	aOR (95% CI)	*p*-value	aOR (95% CI)	*p*-value	aOR (95% CI)	*p*-value
Prenatal &/or postpartum anxiety*	1.08 (0.77–1.53)	0.642	1.07 (0.82–1.40)	0.618	1.00 (0.80–1.26)	0.990
Preterm	1.00 (0.52–1.92)	0.997	-**		-	
Parity	1.73 (1.27–2.36)	0.000	1.03 (0.79–1.34)	0.814	1.44 (1.18–1.75)	0.000
Born Canada	1.57 (1.03–2.39)	0.034	1.74 (1.25–2.42)	0.001	1.48 (1.14–1.93)	0.003
High income	0.43 (0.28–0.68)	0.000	0.65 (0.48–0.90)	0.008	0.71 (0.54–0.93)	0.014
Moves	0.44 (0.20–0.98)	0.044	0.84 (0.56–1.29)	0.431	1.32 (1.03–1.67)	0.026
***Interaction terms***						
High income X Moves***	3.80 (1.60–9.02)	0.002	-		-	
Moves None: High Income versus Low Income	0.43 (0.28–0.67)	0.000	-		-	
Moves > = 1: High Income versus Low Income	1.63 (0.77–3.45)	0.20	-		-	
Moves X Preterm***	2.96 (1.06–8.24)	0.038	-		-	
Moves None: Preterm versus Not Preterm	1.00 (0.52–1.92)	1.000	-		-	
Moves > = 1: Preterm versus Not Preterm	2.96 (1.33–6.57)	0.001	-		-	
Parity X Moves***	-		1.94 (1.11–3.40)	0.021	-	
Moves None: Multiparous versus Primiparous	-		1.03 (0.79–1.34)	0.810	-	
Moves > = 1: Multiparous versus Primiparous	-		2.00 (1.22–3.29)	0.010	-	

* Prenatal (< 25 weeks gestation) and/or postpartum (4 months post-partum) anxiety

** Variables were not statistically significant in the model and therefore were excluded during the backward elimination process

*** Interaction between explanatory variables

As with the depression model, certain covariates were associated with prolonged delay in the anxiety model. Children of mothers born in Canada had higher odds of prolonged delay for DTaP-IPV-Hib (OR 1.57, 95%CI 1.03–2.39) MMR/MMRV (OR 1.74, 95% CI 1.25–2.42), and all vaccine combined (OR 1.48, 95% CI 1.14–1.93, Table 5) than those with mothers born elsewhere.

Notably for DTaP-IPV-Hib vaccine, interaction between some covariates was observed (Table 5, Supplementary Figs. 6–8). In the anxiety model, among mothers not moving households, children of high-income mothers had lower odds of prolonged delay of DTaP-IPV-HiB immunization compared to those of low-income mothers (OR: 0.43, 95% CI 0.28–0.67, Table 5, Supplementary Fig. 6). In the anxiety model, preterm babies had higher odds of prolonged DTaP delay than full term babies among mothers experiencing one or more moves (OR: 2.96, 95% CI 1.33–6.57, Table 5, Supplementary Figure Fig. 7).

Interaction between parity and household moves was observed for MMR/MMRV immunization as well. In the anxiety model, among mothers experiencing multiple moves, children of multiparous mothers had higher odds of prolonged delay compared to those of primiparous mothers (OR: 2.0, 95% CI 1.22–3.29, Table 5, Supplementary Fig. 8).

### Sensitivity analysis

We conducted a sensitivity analysis to assess depression and anxiety only prenatally and only postpartum with no change in the relationships i.e. it did not impact the likelihood of a child having prolonged delayed for DTaP-IPV-Hib and MMR/MMRV, and all vaccines combined (Supplementary Tables 4–6).

## Discussion

This study assessed the role of both prenatal and/or postpartum depression and anxiety on the relationship with timeliness of routine childhood immunization. Interestingly, prenatal and/or postpartum depression was not associated with prolonged vaccine delay for a multi-dose vaccine (i.e., DTaP-IPV-Hib), a single dose vaccine (i.e., MMR/MMRV), nor for all five routine childhood vaccines combined. Previous studies have reported contradictory findings on the effect of maternal mental depression and childhood immunization. For instance, a recent study in the UK reported that children with mothers having depression between one year prior to child’s birth up to the age of two years and five years had reduced likelihood of receiving DTaP/IPV/Hib and MMR vaccines [14]. Likewise, a study done in the USA reported that children whose mothers had depressive symptoms in the 2–4 months’ post-partum period were less likely to receive up-to-date immunization at 24 months for MMR, DTP, and polio vaccines [10]. Similarly, Australian children of mothers experiencing depression were more likely to be immunized late or not at all [12]. In contrast, consistent with our study findings, some studies in the USA and Zambia reported no association between maternal depression and childhood immunization status [3, 11, 13].

Likewise, prenatal and/or postpartum anxiety had no association with prolonged delay, for all five routine childhood vaccines combined, or for single-dose (i.e., MMR/MMRV) and multi-dose (i.e., DTaP-IPV-Hib) vaccines. Previous studies revealed contradictory findings on the association of maternal anxiety with childhood immunization. In one study, children whose mothers had anxiety symptoms were less likely to receive complete immunization at two years and five years for DTaP/IPV/Hib and MMR vaccines [14]. In another study, maternal anxiety was associated with incomplete immunization for all vaccines among children younger than three years old [4]. Likewise, another study found that children of mothers experiencing postpartum anxiety were more likely to be immunized late or not at all [12]. Consistent with our study findings, a US study found no association between mothers with perinatal anxiety and childhood immunization at 8 months of age [3].

Comparability with previous studies examining maternal depression and anxiety and routine childhood immunization is limited due to differences in study settings, health system, depression and anxiety measurement or definitions of outcome variables. One possible reason behind lack of association between vaccine delay and either depression or anxiety in our setting could be the increased use of primary health care services (family physicians/public health nurses) by mothers for prenatal and/or postpartum depression and anxiety symptoms, as has been recognized elsewhere [32]. Mothers receiving mental care support and treatment during prenatal and/or postpartum period could resolve mental health challenges and might have a positive impact on the wellbeing of mother and babies, or the vaccine delivery system is adequate to reach these children despite maternal challenges; however, these assumptions should be investigated in future studies.

Other than depression and anxiety, other factors were associated with delayed childhood immunization status. Children from mothers born in Canada were more likely to have prolonged delay for all vaccines compared to mothers born elsewhere. This finding was consistent with a previous study that reported children from mothers born in Canada, versus immigrant mothers, were less likely to be completely immunized [33]. In line with previous studies [25, 34], we found that a greater number of household moves and low income increased the likelihood of prolonged delay for all vaccines. For example, low income and one or more household moves could influence vaccine compliance, as parents have to orient themselves to the new neighborhood, clinics, healthcare provider, as well as resources.

Although an independent effect of maternal depression and/or anxiety on the timeliness of childhood immunization was not evident, a number of other factors interacted to increase the likelihood of a child having prolonged delay for immunization. For example, joint presence of two or more factors such as one or more household moves, born in Canada, preterm child, multiparous mother, and income was associated with the timeliness of childhood immunization. There is growing recognition that constellations of factors (i.e. multiple overlapping factors such as those stated above) can amplify barriers to immunization, and other health services, beyond the additive impact of the individual factors [29, 34]. This concept, referred to as ‘intersectionality’ should be considered further in future immunization coverage research [35, 36].

### Strengths and limitations

This study was able to link administrative health and survey data; the former aided in obtaining accurate and complete outcome data. The use of validated tools for measuring depression and anxiety at multiple time points during the study was another strength. However, there are some noteworthy limitations of this study. Firstly, this study cohort was representative of urban families in Canada, which may over-represent those with higher socioeconomic status (SES). Thus, the findings may not be generalizable to lower SES families. Secondly, maternal depression and anxiety assessment was based on maternal self-reporting, which could be prone to measurement error and self-report bias. In addition, these scales represent depression and anxiety symptomatology, and not clinical diagnoses. Further, we did not have accurate information on treatment or support that may have been received by mothers during or subsequent to the perinatal period. Such information might help to understand the lack of associations found between maternal mental health symptoms and child vaccine outcomes in the present study.

## Conclusion

This study suggests that prenatal and/ or postpartum maternal depression and anxiety was not associated with timeliness of infants’ immunization. Health care providers can be reassured that maternal depression and anxiety do not influence vaccine adherence. Adherence to vaccine schedules was more difficult among Canadian born mothers and those who experienced one or more household moves and of low income. Provision of information about location of immunization services may assist mothers who are relocating in vaccine adherence, particularly those with income insecurity.

### Electronic supplementary material

Below is the link to the electronic supplementary material.

Supplementary Material 1

## Data Availability

The data set from this study is held securely in coded and de-identified form at Alberta Health Services (AHS). Although data sharing agreements prohibit AHS from making the data set publicly available, access may be granted to those who meet pre-specified criteria for confidential access. Please contact research.administration@ahs.ca for more information.
