# Supplementary material for: Impact of maternal depression and anxiety on immunization status of children: a prospective cohort study
Source: Arch Public Health. 2024 Jun 17;82:89. doi: 10.1186/s13690-024-01323-3 (PMC11181576; doi:10.1186/s13690-024-01323-3)
Supplement: Supplementary file 1 — Supplementary Material 1 [file 13690_2024_1323_MOESM1_ESM.docx]

**SUPPLEMENTARY FILE**

Total participants in the All Our Babies Cohort

N= 3387

Consented to linkage and provided Provincial Health Number (PHN)

N= 2855

Successfully linked to Vaccination Records

N= 2762

Invalid PHN

n=2

Missing child information

n=13

Recurrent births

n=31

Moved out of region

n=46

**Supplementary Figure 1. Total sample derived from linkage of the All Our Families cohort with public health immunization records in Calgary, Alberta (2008-2010)**

#
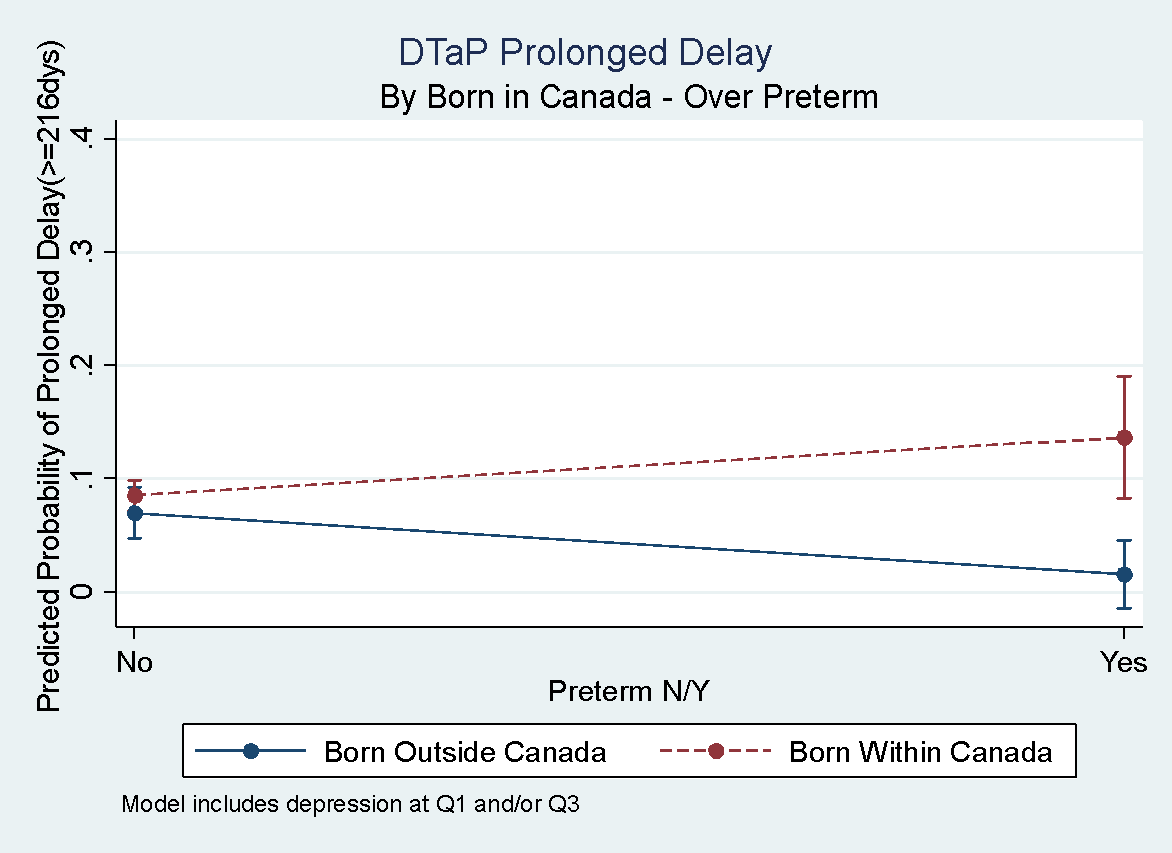


**Supplementary Figure 2**. **Effect of interaction between mother born in Canada and preterm status of child on prolonged delay in DTaP-IPV-Hib immunization for prenatal &/or postpartum *depression* model among children in Calgary, Alberta (N=2762)**

**
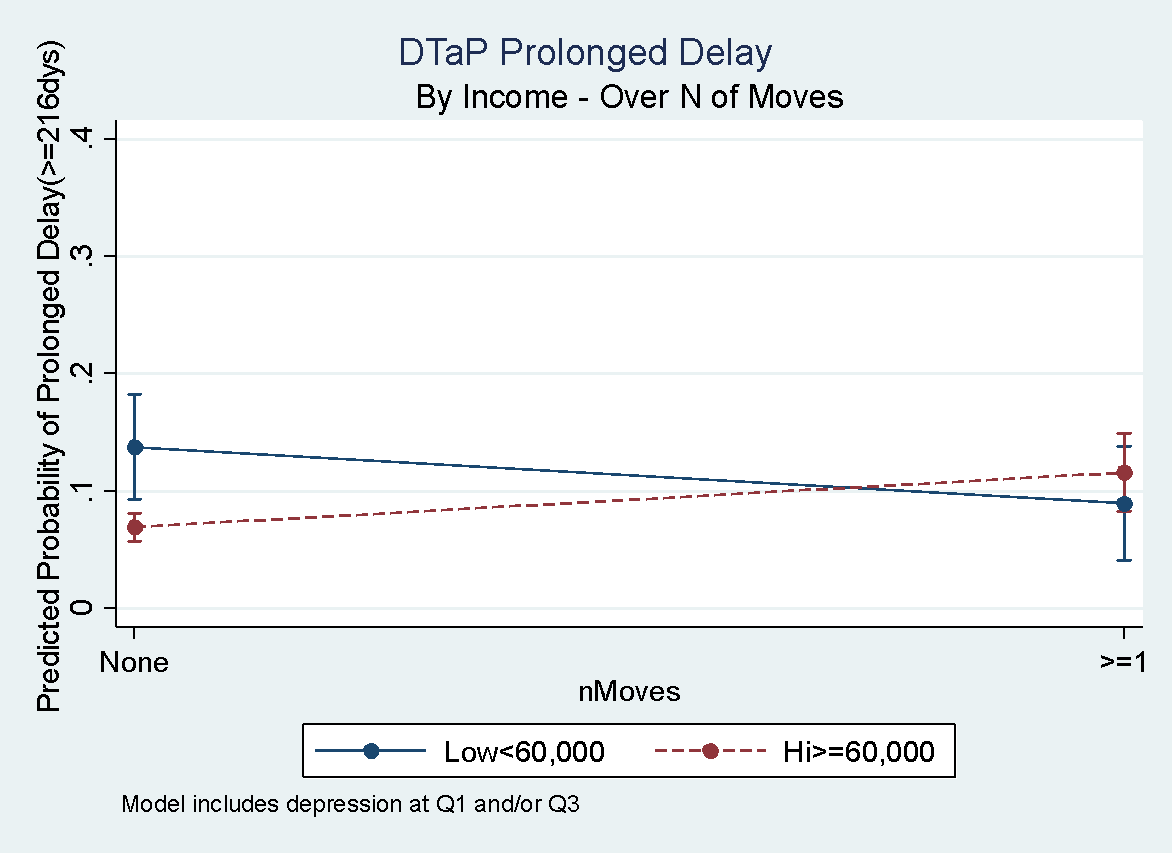
**

**Supplementary Figure 3.** **Effect of interaction between income and household moves on prolonged delay in DTaP-IPV-Hib immunization for prenatal &/or postpartum *depression* model among children in Calgary, Alberta (N=2762)**

**
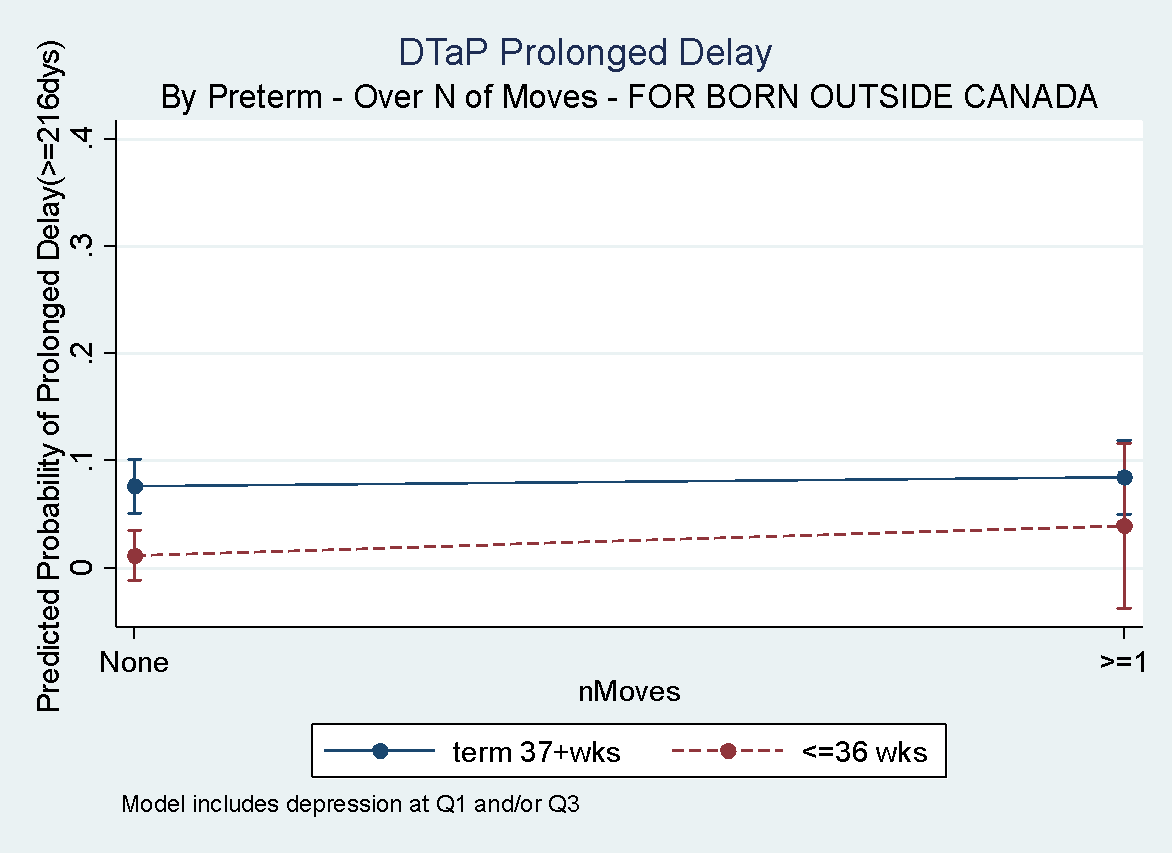
**

**Supplementary Figure 4. Effect of interaction between preterm status of child and household moves on prolonged delay in DTaP-IPV-Hib immunization for prenatal &/or postpartum *depression* model among children in Calgary, Alberta (N=2762) whose mothers were born outside Canada**

**
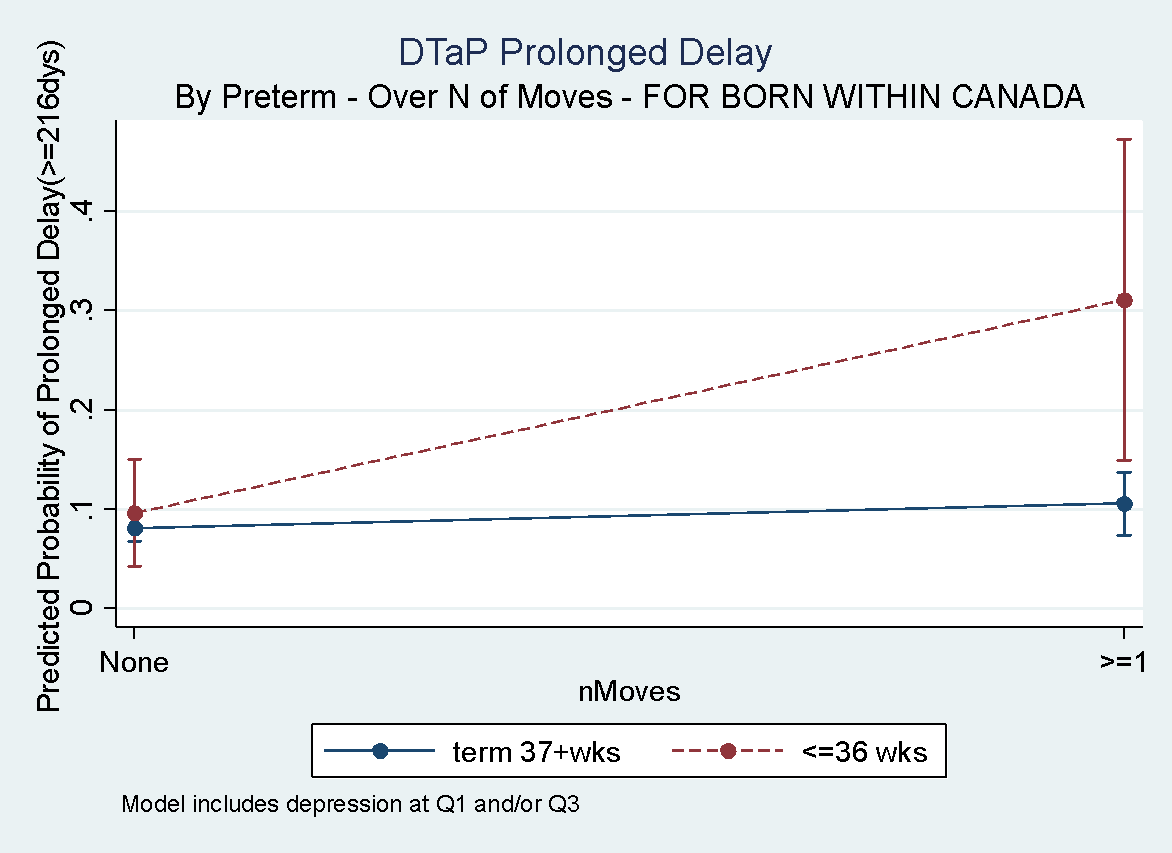
**

**Supplementary Figure 5. Effect of interaction between preterm status of child and household moves on prolonged delay in DTaP-IPV-Hib immunization for prenatal &/or postpartum *depression* model among children in Calgary, Alberta (N=2762) whose mothers were born in Canada**


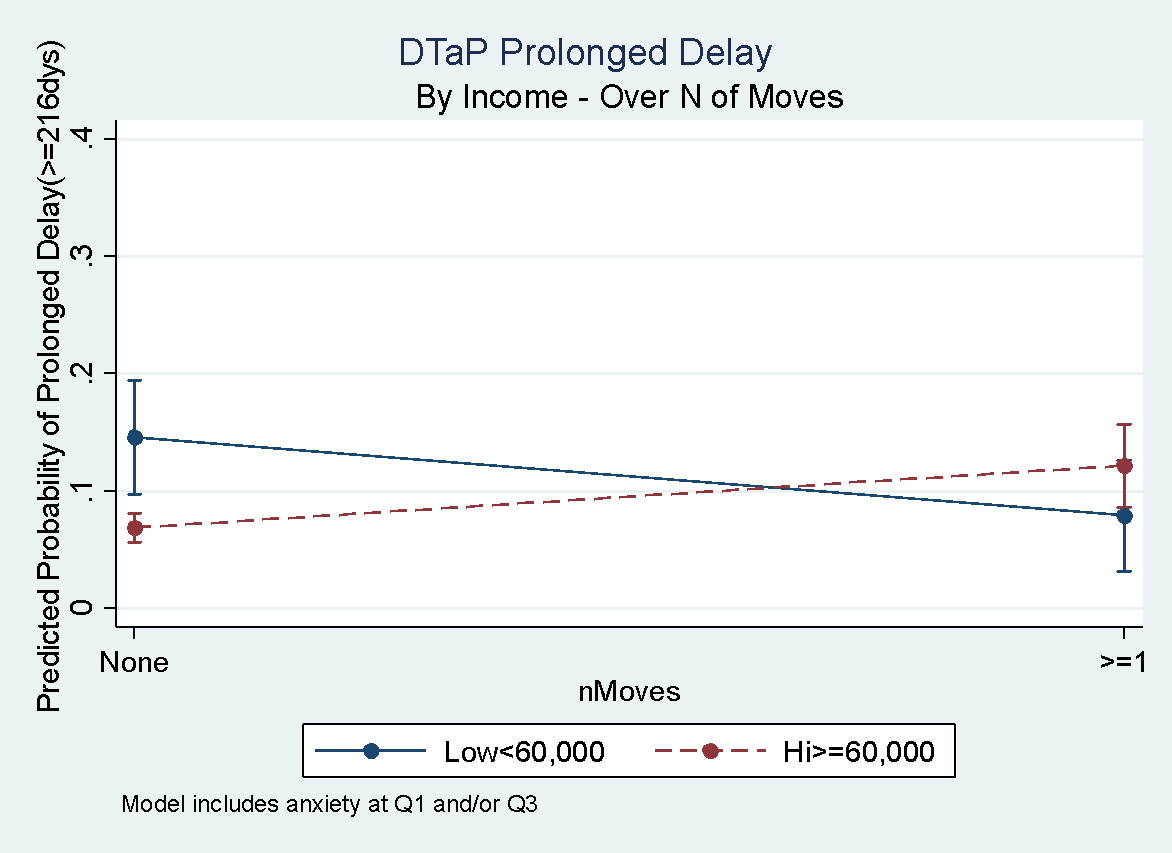


**Supplementary Figure 6. Effect of interaction between income and household moves on prolonged delay in DTaP-IPV-Hib immunization for prenatal &/or postpartum *anxiety* model among children in Calgary, Alberta (N=2762)**


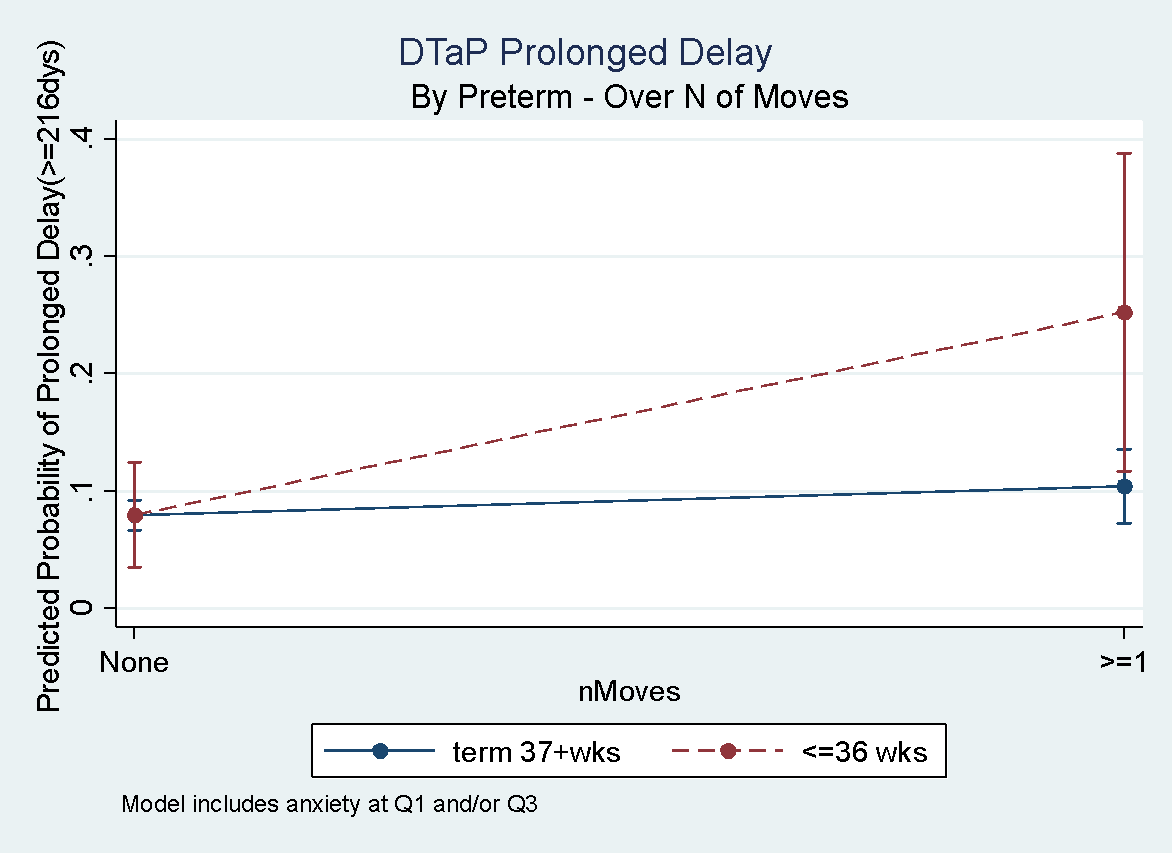


**Supplementary Figure 7. Effect of interaction between preterm status of child and household moves on prolonged delay in DTaP-IPV-Hib immunization for prenatal &/or postpartum *anxiety* model among children in Calgary, Alberta (N=2762)**


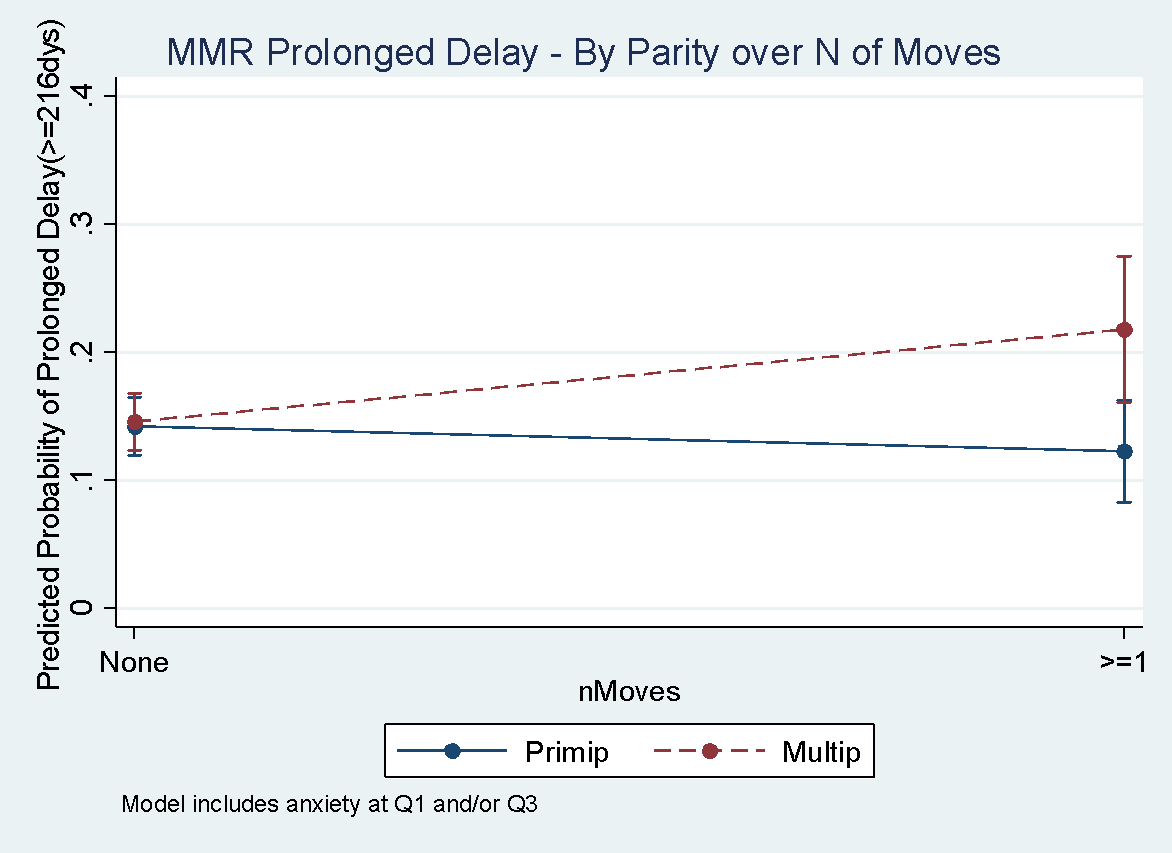


**Supplementary Figure 8. Effect of interaction between parity and household moves on prolonged delay in MMR immunization for prenatal &/or postpartum *anxiety* model among children in Calgary, Alberta (N=2762)**

**Supplementary Table 1: Comparison of proportion and percentage of sample who had prolonged delay of all vaccines for each level of exposure variables and covariates**

| **Variables** | **Proportion with Prolonged Delay (n/N)** | **Percentage with Prolonged Delay (%)** | **P-value*** |
| --- | --- | --- | --- |
| ***Depression***  Prenatal and/or postpartum depression | 63/266 | 23.68 | 0.767 |
| No prenatal and/or postpartum depression | 531/2321 | 22.88 |  |
| **Anxiety**  Prenatal and/or postpartum anxiety | 136/581 | 23.41 | 0.756 |
| No prenatal and/or postpartum anxiety | 422/1852 | 22.79 |  |
| ***Parity***  Multi-parity | 352/1366 | 25.77 | 0.001 |
| Primiparous | 279/1365 | 20.44 |  |
| ***Marital status***  Married/ common law | 602/2613 | 23.04 | 0.675 |
| Other | 29/135 | 21.48 |  |
| ***Born in Canada***  Yes | 515/2147 | 23.99 | 0.027 |
| No | 119/604 | 19.70 |  |
| ***Post-secondary education***  Yes | 557/2461 | 22.63 | 0.103 |
| No | 77/286 | 26.92 |  |
| ***Income***  High Income ≥ $60,000 | 486/2218 | 21.91 | 0.005 |
| Low Income <60,000 | 125/445 | 28.09 |  |
| ***Social support***  High social support | 464/2010 | 23.08 | 0.765 |
| Low social support | 135/570 | 23.68 |  |
| ***Move***  ≥1 household moves | 139/521 | 26.68 | 0.027 |
| None | 463/2092 | 22.13 |  |
| ***Preterm***  ≤36 wks | 45/208 | 21.63 | 0.658 |
| Term ≥37 wks | 548/2385 | 22.98 |  |

*** Pearson’s Chi-squared test

**Supplementary Table 2: Comparison of proportion and percentage of sample who had prolonged delay of DTaP vaccine for each level of exposure variables and covariates**

| **Variables** | **Proportion with Prolonged Delay (n/N)** | **Percentage with Prolonged Delay (%)** | **P-value*** |
| --- | --- | --- | --- |
| ***Depression***  Prenatal and/or postpartum depression | 30/266 | 11.28 | 0.092 |
| No prenatal and/or postpartum depression | 191/2321 | 8.23 |  |
| ***Anxiety***  Prenatal and/or postpartum anxiety | 56/581 | 9.64 | 0.301 |
| No prenatal and/or postpartum anxiety | 153/1852 | 8.26 |  |
| ***Parity***  Multi-parity | 144/1366 | 10.54 | 0.002 |
| Primiparous | 97/1365 | 7.11 |  |
| ***Marital status***  Married/ common law | 231/2613 | 8.84 | 0.566 |
| Other | 10/135 | 7.41 |  |
| ***Born in Canada***  Yes | 196/2147 | 9.13 | 0.246 |
| No | 46/604 | 7.62 |  |
| ***Post-secondary education***  Yes | 206/2461 | 8.37 | 0.017 |
| No | 36/286 | 12.59 |  |
| ***Income***  High Income ≥ $60,000 | 173/2218 | 7.80 | 0.001 |
| Low Income <60,000 | 57/445 | 12.81 |  |
| ***Social support***  High social support | 174/2010 | 8.66 | 0.565 |
| Low social support | 45/570 | 7.89 |  |
| ***Move***  ≥1 household moves | 58/521 | 11.13 | 0.014 |
| None | 163/2092 | 7.79 |  |
| ***Preterm***  ≤36 wks | 22/208 | 10.58 | 0.249 |
| Term ≥37 wks | 197/2385 | 8.26 |  |

* Pearson’s Chi-squared test

**Supplementary Table 3: Comparison of proportion and percentage of sample who had prolonged delay of MMR vaccine for each level of exposure variables and covariates**

| **Variables** | **Proportion with Prolonged Delay (n/N)** | **Percentage with Prolonged Delay (%)** | **P-value*** |
| --- | --- | --- | --- |
| ***Depression***  Prenatal and/or postpartum depression | 40/266 | 15.04 | 0.895 |
| No prenatal and/or postpartum depression | 342/2321 | 14.74 |  |
| ***Anxiety***  Prenatal and/or postpartum anxiety | 89/581 | 15.32 | 0.638 |
| No prenatal and/or postpartum anxiety | 269/1852 | 14.52 |  |
| ***Parity***  Multi-parity | 218/1366 | 15.96 | 0.097 |
| Primiparous | 187/1365 | 13.70 |  |
| ***Marital status***  Married/ common law | 383/2613 | 14.66 | 0.600 |
| Other | 22/135 | 16.30 |  |
| ***Born in Canada***  Yes | 340/2147 | 15.84 | 0.004 |
| No | 67/604 | 11.09 |  |
| ***Post-secondary education***  Yes | 360/2461 | 14.63 | 0.416 |
| No | 47/286 | 16.43 |  |
| ***Income***  High Income ≥ $60,000 | 314/2218 | 14.16 | 0.021 |
| Low Income <60,000 | 82/445 | 18.43 |  |
| ***Social support***  High social support | 302/2010 | 15.02 | 0.947 |
| Low social support | 85/570 | 14.91 |  |
| ***Move***  ≥1 household moves | 88/521 | 16.89 | 0.151 |
| None | 301/2092 | 14.39 |  |
| ***Preterm***  ≤36 wks | 29/208 | 13.94 | 0.714 |
| Term ≥37 wks | 355/2385 | 14.88 |  |

* Pearson’s Chi-squared test

**Supplementary Table 4. Final logistic regression multivariable model depicting association between maternal anxiety and depression at two different time points i.e. prenatal (< 25 weeks gestation) and postpartum (4 months postpartum), and prolonged delay in all routine childhood immunizations among children in Calgary, Alberta (N=2762)**

| **Model** | **Outcome** | **Exposure and covariates** | **Odds ratio** | **95% CI** | **P-value** |
| --- | --- | --- | --- | --- | --- |
| Prenatal depression | Prolonged delay | Prenatal depression (exposure) | 1.27 | 0.88 - 1.83 | 0.20 |
|  |  | Born in Canada | 1.39 | 1.09 - 1.77 | 0.01 |
|  |  | Parity | 1.35 | 1.12 - 1.63 | 0.00 |
|  |  | High income | 0.60 | 0.44 - 0.82 | 0.00 |
|  |  | Household moves | 0.75 | 0.47 - 1.21 | 0.24 |
|  |  | High income X household moves* | 1.95 | 1.14 - 3.36 | 0.02 |
| Postpartum depression | Prolonged delay | Postpartum depression (exposure) | 0.54 | 0.30 - 0.98 | 0.04 |
|  |  | parity | 1.37 | 1.13 - 1.66 | 0.00 |
|  |  | Born Canada | 1.37 | 1.08 - 1.75 | 0.01 |
|  |  | Moves | 0.76 | 0.47 - 1.23 | 0.27 |
|  |  | High income | 0.60 | 0.44 - 0.82 | 0.00 |
|  |  | Postpartum depression X moves* | 2.98 | 1.15 - 7.74 | 0.03 |
|  |  | High income X moves* | 1.82 | 1.06 - 3.13 | 0.03 |
| Prenatal anxiety | Prolonged delay | Prenatal anxiety (exposure) | 1.04 | 0.80 - 1.36 | 0.75 |
|  |  | parity | 1.39 | 1.15 - 1.69 | 0.00 |
|  |  | Born Canada | 1.45 | 1.12 - 1.87 | 0.00 |
|  |  | High income | 0.57 | 0.42 - 0.78 | 0.00 |
|  |  | Moves | 0.77 | 0.47 - 1.25 | 0.29 |
|  |  | High income X moves* | 1.90 | 1.09 - 3.31 | 0.02 |
| Postpartum anxiety | Prolonged delay | Postpartum anxiety (exposure) | 0.98 | 0.75 - 1.28 | 0.87 |
|  |  | Parity | 1.38 | 1.14 - 1.68 | 0.00 |
|  |  | Born Canada | 1.39 | 1.08 - 1.78 | 0.01 |
|  |  | High income | 0.59 | 0.43 - 0.82 | 0.00 |
|  |  | Moves | 0.79 | 0.48 - 1.28 | 0.33 |
|  |  | High income X moves* | 1.96 | 1.13 - 3.40 | 0.02 |

* Interaction between explanatory variables

**Supplementary Table 5. Final logistic regression multivariable model depicting association between maternal anxiety and depression at two different time points i.e., prenatal (< 25 weeks gestation) and postpartum (4 months post-partum), and prolonged delay in DTaP-IPV-Hib immunization among children in Calgary, Alberta (N=2762)**

| **Model** | **Outcome** | **Exposure and covariates** | **Odds ratio** | **95% CI** | **P-value** |
| --- | --- | --- | --- | --- | --- |
| Prenatal depression | Prolonged delay | Prenatal depression (exposure) | 1.53 | 0.92 - 2.56 | 0.10 |
|  |  | Parity | 1.61 | 1.20 - 2.16 | 0.00 |
|  |  | Born Canada | 1.50 | 1.02 - 2.21 | 0.04 |
|  |  | High income | 0.47 | 0.31 - 0.72 | 0.00 |
|  |  | Moves | 0.51 | 0.24 - 1.06 | 0.07 |
|  |  | Preterm | 0.92 | 0.48 - 1.75 | 0.80 |
|  |  | High income X moves* | 3.13 | 1.39 - 7.03 | 0.01 |
|  |  | Moves X preterm* | 2.88 | 1.04 - 7.95 | 0.04 |
| Postpartum depression | Prolonged delay | Postpartum depression (exposure) | 1.15 | 0.63 - 2.11 | 0.65 |
|  |  | Parity | 1.60 | 1.19 - 2.15 | 0.00 |
|  |  | High income | 0.49 | 0.32 - 0.75 | 0.00 |
|  |  | Moves | 0.53 | 0.25 - 1.10 | 0.09 |
|  |  | Preterm | 0.93 | 0.49 - 1.76 | 0.82 |
|  |  | High income X moves* | 3.00 | 1.34 - 6.73 | 0.01 |
|  |  | Moves X preterm* | 2.91 | 1.06 - 8.01 | 0.04 |
| Prenatal anxiety | Prolonged delay | Prenatal anxiety (exposure) | 38.13 | 1.89 - 769.54 | 0.02 |
|  |  | Parity | 1.86 | 1.36 - 2.55 | 0.00 |
|  |  | Maternal age at delivery | 0.99 | 0.96 - 1.03 | 0.71 |
|  |  | Moves | 0.54 | 0.24 - 1.20 | 0.13 |
|  |  | High income | 0.49 | 0.32 - 0.76 | 0.00 |
|  |  | Preterm | 0.91 | 0.47 - 1.74 | 0.77 |
|  |  | Prenatal anxiety X maternal age at delivery * | 0.89 | 0.81 - 0.99 | 0.02 |
|  |  | Prenatal anxiety X moves* | 0.20 | 0.06 - 0.67 | 0.01 |
|  |  | High income X moves* | 3.25 | 1.37 - 7.73 | 0.01 |
|  |  | Moves X preterm* | 3.83 | 1.35 - 10.86 | 0.01 |
| Postpartum anxiety | Prolonged delay | Postpartum anxiety (exposure) | 1.28 | 0.87 - 1.88 | 0.20 |
|  |  | Parity | 1.64 | 1.21 - 2.22 | 0.00 |
|  |  | Born Canada | 1.28 | 0.85 - 1.93 | 0.24 |
|  |  | Preterm | 0.45 | 0.04 - 4.54 | 0.50 |
|  |  | Education | 1.06 | 0.62 - 1.81 | 0.84 |
|  |  | High income | 0.49 | 0.31 - 0.76 | 0.00 |
|  |  | Moves | 0.51 | 0.23 - 1.10 | 0.09 |
|  |  | Born Canada X preterm* | 9.41 | 1.12 - 78.96 | 0.04 |
|  |  | educPS X preterm* | 0.24 | 0.06 - 0.92 | 0.04 |
|  |  | High income X moves* | 3.28 | 1.40 - 7.67 | 0.01 |
|  |  | Moves X preterm* | 3.02 | 1.03 - 8.85 | 0.04 |

* Interaction between explanatory variables

**Supplementary Table 6. Final logistic regression multivariable model depicting association between maternal anxiety and depression at two different time points i.e., prenatal (< 25 weeks gestation) and postpartum (4 months post-partum), and prolonged delay in MMR immunization among children in Calgary, Alberta (N=2762)**

| **Exposure** | **Outcome** | **Final model variables** | **Odds ratio** | **95% CI** | **P-value** |
| --- | --- | --- | --- | --- | --- |
| Prenatal depression | Prolonged delay | Prenatal depression (exposure) | 1.50 | 1.03- 2.20 | 0.04 |
|  |  | Born Canada | 1.68 | 1.26- 2.25 | 0.00 |
|  |  | Income high | 0.69 | 0.52- 0.91 | 0.01 |
| Postpartum depression | Prolonged delay | Postpartum depression (exposure) | 0.63 | 0.35- 1.13 | 0.12 |
|  |  | Born Canada | 1.57 | 1.16- 2.11 | 0.00 |
|  |  | High income | 0.67 | 0.50- 0.89 | 0.01 |
| Prenatal anxiety | Prolonged delay | Prenatal anxiety (exposure) | 1.13 | 0.83- 1.53 | 0.45 |
|  |  | Born Canada | 1.72 | 1.25- 2.36 | 0.00 |
|  |  | High income | 0.65 | 0.48- 0.89 | 0.01 |
|  |  | Parity | 1.02 | 0.79- 1.32 | 0.86 |
|  |  | Moves | 0.82 | 0.54- 1.23 | 0.33 |
|  |  | Parity X moves* | 1.95 | 1.12- 3.40 | 0.02 |
| Postpartum anxiety | Prolonged delay | Postpartum anxiety (exposure) | 1.01 | 0.74- 1.38 | 0.94 |
|  |  | Born Canada | 1.60 | 1.18- 2.18 | 0.00 |
|  |  | Income high | 0.68 | 0.50- 0.91 | 0.01 |

* Interaction between explanatory variables
